# Community-based interventions for obesity prevention: lessons learned by Australian policy-makers

**DOI:** 10.1186/1756-0500-5-20

**Published:** 2012-01-10

**Authors:** Michelle M Haby, Rebecca Doherty, Nicky Welch, Vicky Mason

**Affiliations:** 1 Prevention & Population Health Branch, Victorian Government Department of Health, 50 Lonsdale St, Melbourne, Victoria 3000, Australia; 2 Centre for Health Policy, Programs and Economics; School of Population Health, University of Melbourne, 207 Bouverie St, Carlton, Victoria 3053, Australia; 3 La Trobe Rural Health School, Faculty of Health Sciences, P.O. Box 199, Bendigo, Victoria 3552, Australia

## Abstract

**Background:**

Interest in community-based interventions (CBIs) for health promotion is increasing, with a lot of recent activity in the field. This paper aims, from a state government perspective, to examine the experience of funding and managing six obesity prevention CBIs, to identify lessons learned and to consider the implications for future investment. Specifically, we focus on the planning, government support, evaluation, research and workforce development required.

**Methods:**

The lessons presented in this paper come from analysis of key project documents, the experience of the authors in managing the projects and from feedback obtained from key program stakeholders.

**Results:**

CBIs require careful management, including sufficient planning time and clear governance structures. Selection of interventions should be based on evidence and tailored to local needs to ensure adequate penetration in the community. Workforce and community capacity must be assessed and addressed when selecting communities. Supporting the health promotion workforce to become adequately skilled and experienced in evaluation and research is also necessary before implementation.

Comprehensive evaluation of future projects is challenging on both technical and affordability grounds. Greater emphasis may be needed on process evaluation complemented by organisation-level measures of impact and monitoring of nutrition and physical activity behaviours.

**Conclusions:**

CBIs offer potential as one of a mix of approaches to obesity prevention. If successful approaches are to be expanded, care must be taken to incorporate lessons from existing and past projects. To do this, government must show strong leadership and work in partnership with the research community and local practitioners.

## Background

Recent trends in health promotion emphasise community-based (or whole-of-community) programs as an important strategy for achieving population-level change in risk factors and health. Internationally and in Australia, there has been a shift from individually-focused interventions to a socio-ecological approach that looks beyond the individual to the environmental factors that impact on health and wellbeing [1,2]. This approach encourages action at many levels, including the interpersonal, organisational, community and policy [3].

While the definition of community-based interventions (CBIs) is not widely agreed, Merzel and D'Afflitti offer a framework that includes six core elements: 1) integrated and comprehensive; 2) involve a range of locations; 3) employ multiple interventions; 4) include multiple individuals, organisations, groups; 5) involve the community in planning, implementation, management and evaluation; and 6) include multiple individual-level intervention strategies [1].

To date, evidence for the effectiveness of CBIs in improving health outcomes varies. While there is some evidence for improved mental health [4], increased participation in physical activity [5], increased fruit and vegetable consumption [6-8] and reduction in health inequalities [9], there is equivocal evidence to support a change in alcohol consumption [10,11]. In general, the impact of CBIs has been modest (with reduction in risk factors of between 5 and 15% at most) [4-9], with the exception of a number of HIV prevention programs that have shown larger impacts [1].

There is a great deal of recent activity in the field of CBIs, especially in relation to obesity prevention, with a number of current CBIs showing promise. An Australian trial, Colac Be Active Eat Well (BAEW, 2002-2006) targeted at 4-12 year olds, showed significantly lower increases in body weight (≈1 kg) and waist circumference (≈3 cm) in participating children when compared to controls [12]. Importantly, this study showed no increase in health inequalities or psychological harm. Another Australian trial targeted at children under 5 years of age and their families (Romp & Chomp, 2004-2008) showed a significant reduction in the prevalence of overweight and obesity in the intervention community [13].

As well as Colac BAEW and Romp & Chomp, the Victorian State Government has jointly sponsored several other large community-based interventions, including Fun 'n' Healthy in Moreland (2004-2010) and It's Your Move! (2004-2010), and has fully funded six 'Go for your life' Health Promoting Communities: Being Active and Eating Well (HPC:BAEW) projects across the state. Together, these projects represent a significant investment in planning, implementation and research as well as a large investment of personnel time (government, research, organisations involved and other key stakeholders) and in-kind support. In addition, there is significant work and investment underway in Victoria [14], in other states [15], at a federal government level [16,17] and internationally [18].

Nonetheless, there is a gap in the literature on the lessons from within government as funder and policy maker. Another gap concerns the implications of these projects and lessons for future "roll-out" or mainstreaming. Most of the literature in this area is written from the perspective of researchers [19,20]. Any significant investment in this area will come from government who will have an important role in defining what and how these initiatives will be delivered and expected outcomes. Thus, it is important that lessons from current projects are incorporated into the peer-reviewed literature and debate encouraged, ensuring that any future investment results in the best value for money and best health outcomes for all involved.

The aims of this paper are: to reflect upon the experience of overseeing a number of CBIs, specifically the 6 HPC:BAEW projects; to identify lessons learned; and to consider the implications for future investment. In particular, we focus on the planning, government support, evaluation, research and workforce development required to support future investment in CBIs. This paper is unique in that it is written from the perspective of policy-makers and funders and is based upon the experience of staff (including some with research expertise) within a state government health department managing a program of work on CBIs for obesity prevention.

### The six HPC:BAEW projects

The HPC:BAEW projects (originally funded in 2006 for 4 years) were initiated by government to build on the experience and success from the Colac BAEW program. The HPC:BAEW program (Table 1) was of larger scale (involving six different communities), incorporated greater complexity (including different age groups, rural and metropolitan areas, culturally and socioeconomically diverse populations, different governance and management structures) and was intended to be the next step in the potential state-wide roll-out of obesity prevention CBIs. The program had a budget of $AUD 3.6 M for implementation and $AUD 0.74 M for evaluation.

**Table 1:** Summary of HPC:BAEW communities

Intervention Community	Community characteristics	Primary target group	Secondary target group	Budget	Funding timeframe
Cardinia Shire Council/South-East Healthy Communities Partnership	Urban; high level of socio-economic disadvantage	Primary school-aged children, 5-12 years	Families, carers, older adults and seniors	$637,000	Apr 2007-June 2010

Kingston Bayside PCP	Urban; culturally and linguistically diverse; high level of socio-economic disadvantage	Children 0-12 years	Families, carers, older adults and seniors	$637,000	Apr 2007-June 2010

Campaspe PCP	Rural; high level of socio-economic disadvantage; high proportion of young people	Secondary school-aged children, 12-18 years	Older adults	$637,000	Apr 2007-June 2010

Westbay PCP (HealthWest Partnership)	Urban; culturally and linguistically diverse; high level of socio-economic disadvantage	Secondary school-aged children, 12-18 years	Young people newly arrived from overseas	$637,000	Apr 2007-June 2010

Southern Grampians and Glenelg PCP (SGG)	Rural; culturally homogeneous; ageing population; high level of socio-economic disadvantage	Working adults	Wider community	$637,000	Apr 2007-June 2010

Wathaurong Aboriginal Co-operative	Regional; Aboriginal community; high level of socio-economic disadvantage	Whole of community*		$392,000	Oct 2007-June 2010

* This later moved to a heavy emphasis on increasing physical activity in children and adolescents

Projects were all located in regions of socioeconomic disadvantage, including rural and metropolitan locations ranging in population size from approximately 13,000-35,000 people (compared to approximately 11,000 covered by Colac BAEW) and varying in the level of cultural and socioeconomic diversity within their boundaries.

#### Governance and project management

The HPC:BAEW program was jointly funded by two state government departments—the Department of Health (DH) and the Office of Senior Victorians within the Department of Planning and Community Development (DPCD). Five of the projects were coordinated through primary care partnerships (PCPs) but in most cases were managed by a lead agency from within the PCP, such as a local government or health service. (PCPs are voluntary alliances of health service providers working together within a region; they include hospitals, community health, local government and divisions of general practice as core members [21].) The sixth project was situated within a regional Victorian Aboriginal Community-Controlled Health Organisation [22].

Initially, individual governance structures at the local level were overseen by a program-wide Project Advisory Group, with a chair and secretariat from DH. It was comprised of local project managers from the intervention communities and relevant government stakeholders (project manager level), with the state-wide evaluators joining once appointed. A Project Board (with executive level membership for higher-level decision making) and Expert Reference Group (to provide strategic advice) were added later in the program (Table 2).

**Table 2:** Functions and membership of HPC:BAEW governance groups

	Local project teams/governance structures	Project Advisory Group	Project Board	Expert Reference Group
**Functions**	To bring together the members of the community that will implement the strategies.	To ensure the program objectives of the initiatives are being met To review action plans, budgets, evaluation and communication plans To ensure linkages both across initiatives and with other Victorian activity that promotes physical activity and healthy eating To provide feedback and share best practice Capacity Building: sharing resources & strategies for projects/local participants	Recommendation for sign-off by DH and DPCD of project action plans, evaluation plans, communication strategies etc Decision-making regarding funding, project direction, reporting To plan project publications and communications	To provide input into guiding implementation and evaluation of the demonstration projects with a view to shaping the lessons learned from the projects (knowledge transfer and exchange) Capture and dissemination of project findings Higher-level problem-solving

**Membership**	Key local stakeholders including PCP and LGA representation	Project managers, DH, DPCD, state-wide evaluator	DH, DPCD, Project manager representative, PCP representative, ACCHO representative-Executive level group	DH, DPCD, DEECD, 2 × university-based experts in community-based obesity prevention-Executive level group

Abbreviations

*ACCHO *Aboriginal Community-Controlled Health Organisation, *DEECD *Department of Education & Early Childhood Development, *DH *Department of Health, *DPCD *Department of Planning & Community Development, *LGA *Local Government Area, *PCP *Primary Care Partnership

#### Evaluation

The five PCP-based projects were evaluated through a controlled-trial design coordinated by Deakin University [23]. Local process evaluation (including some impact evaluation) was undertaken by each project. Impact evaluation data collected includes environmental audits, strength of partnerships, individual behaviour change and anthropometry from schools, early childhood settings and workplaces for the primary target group only. The Wathaurong project was evaluated separately by Melbourne University, in conjunction with the local project staff, using process and impact evaluation and no control group.

## Methods

The lessons presented in this paper come primarily from analysis of key project documents, from the experience of the authors in managing the projects, and from stakeholder feedback. This stakeholder feedback was obtained through discussion at the Project Advisory Group and from direct communication between stakeholders and DH (written or verbal).

## Results and discussion

### Lessons learned

#### Project/program management

CBIs are complex and require the early establishment of project management systems. While good program management principles should be followed for all programs, the complexity of CBIs (with multiple stakeholder groups) makes this even more critical. Important steps in early project management include clarification of purpose, identification of stakeholders, their needs and expectations, and determination of the eventual program benefits or measures of success [24].

For large interdepartmental projects such as HPC:BAEW, early identification and involvement of all other government stakeholders is crucial to success. Adequate planning time before implementation can provide time for achieving a common understanding of aims and expectations from all key stakeholders. Research shows that partnerships with key stakeholders are one of the critical success factors for CBIs [1,4,5,25]. In the case of HPC:BAEW, better strategic engagement between the relevant government departments (DH, DPCD and DEECD) would likely have resulted in greater clarity of direction and removal of barriers (such as access to schools) before those responsible for implementation were selected and briefed. This engagement takes time initially but would have saved significant time and effort over the course of the project. Input from evaluators in the early planning stages is also very important.

The establishment of clear governance and communication structures is vital early in a project's development. A good governance structure builds clarity of purpose and relationships between all stakeholders, as well as providing clear accountability arrangements [26]. Despite the presence of the Project Advisory Group, it became evident relatively early that lack of clarity and transparency around decision making and governance was affecting program implementation and stakeholder satisfaction. The establishment of a Project Board and an Expert Reference Group later in the program helped to resolve some of these issues by increasing accountability at all levels. Table 2 describes the composition and function of each element of the governance structure.

As well as helping to regain the confidence of stakeholders, the involvement of executive level staff on the Project Board and Expert Reference Group streamlined the development and endorsement of key documents (Table 3). Developing these documents and structures earlier would have had the added advantage of reducing the impact of staff turnover, a significant issue for all long-term projects, as knowledge of the project and continued commitment to it would have been less dependent on any one staff member. A continuous focus on documentation is paramount so as not to lose key lessons learned.

**Table 3:** Key documents to support governance structure

Document	Function
Communication protocol	Outlines a process for communication and decision-making within the program, including details of key program contacts

Roles and responsibilities charter	Outlines roles and responsibilities for all project stakeholders

Variation log	Documents key decisions along the course of the program that deviate from the original program plan

Publication protocol	Provides guidance on issues such as appropriate consultation, authorship and sign-off procedures for those preparing journal publications arising from the program

Risk management strategy	Documents possible risks and mitigation strategies across the program

Reporting templates	Outlines expectations of project reporting, with a strong focus on appropriate reporting of evaluation results

Program plan	Provides an overview of the program and draws together all key project documents so that these can be readily located by staff within the project management team

The importance of opportunities to incorporate lessons learned as a program progresses has also become clear. A set of 'best practice principles' developed by the Collaboration of Community-based Obesity Prevention Sites [27] recommends a planning process for CBIs that considers available evidence and information about the problem and the factors contributing to the problem, as well as use of the best evidence on the effectiveness of interventions. In an ideal world, the lessons from previous projects would be consolidated before new initiatives are planned. In reality, the existence of government funding and election cycles often means that new projects need to be started before evaluation of earlier work is fully complete. HPC:BAEW was informed by early outcomes from the Colac BAEW project as well as other Australian and international CBIs; however, detailed results from the Colac BAEW project were still emerging. To delay future CBIs until analysis of previous work is complete may lead to missed opportunities and would stifle the production of further evidence on intervention effectiveness. Instead we need to better utilise vehicles for capturing learning as projects progress.

#### Evaluation/research

The evidence base for CBIs was not extensive at the time that the HPC:BAEW projects were funded. Thus, it was important to have a strong research and evaluation component to the program. This required the skills of researchers and a strong study design that included a control group. A state-wide evaluator was appointed to conduct an evaluation of the five PCP-based projects, measure the impacts of the program as a whole, and guide the process evaluation. Local projects were responsible for implementation, process evaluation and measurement of some impacts.

Accurately scoping and costing the evaluation was difficult as this was an emerging area of work. This, combined with relatively slow government procurement processes, meant that the appointment of the evaluator was delayed. While local projects, funded through a different mechanism, received funds quickly and were eager to begin implementation, they were hampered by the lack of evaluation support. The reduction in scope of the evaluation also meant that the responsibility for integrating local and state-wide evaluations fell to project management staff within government. This coordination was hampered by a lack of expertise and insufficient staff time.

Over the course of the program it also became apparent that health promotion teams in the communities and within government lacked experience with some aspects of research and evaluation, particularly in relation to ethics procedures, study design and interpretation of data. In retrospect, more time, effort and support was needed to ensure a common understanding of the research methodology and focus at a local level and to obtain ethics approval. Alternatively, a more substantial investment in the evaluation contract (> 15% of total budget) and early appointment of the evaluators would have enabled the state-wide evaluator to provide further support for local evaluation and may also have facilitated workforce development in each of the projects. The impact of scaling up from one to six communities cannot be underestimated and the capacity of evaluators (in terms of workload) must be considered when making decisions about roll-out of interventions state-wide.

Evaluation design must also be considered carefully: individual-level measures of anthropometry and behaviour change, especially when they require opt-in consent for minors, may no longer be feasible due to low response rates and the impact this has upon the validity and generalisability of the results. The baseline response rate for questionnaires and anthropometry measures in HPC:BAEW was less than 40% (and as low as 27% in adolescents), even after a second round of data collection and removal of anthropometry for new participants. Evaluations of other CBIs in Victoria and South Australia have been able to achieve response rates of around 50% [12,20] which, while higher, still raise the issue of possible selection bias and exaggeration of the impact of the intervention. Of course, once the effectiveness of CBIs is known the need for a comprehensive evaluation of such projects may be less and monitoring of impacts will be more appropriate.

#### Interventions

The evidence from the literature shows that effective CBIs require significant penetration or exposure within the community—that is, the proportion of community members who take part in the CBI must be sufficient [25]. Strategies to facilitate this include: the use of an ecological (community-wide) rather than individual approach; partnerships with key stakeholders; and inclusion of multiple interventions with a greater focus on interventions aimed at organisations and policies in addition to individuals and groups [25]. Interventions need to be tailored to address community needs and conditions by providing strategies that are appropriate to all segments of the community and reaching different sub-groups, including disadvantaged populations [25].

This requires adequate resources, which may be challenging when the involvement of the whole community is required. Merzel and D'Afflitti suggest that one of the reasons the HIV programs were successful where CBIs addressing other issues were not was the targeting of relatively small, more homogenous social groups [1]. Other reasons for the success of HIV programs include the nature and degree of infection risk involved, the considerable involvement of the community for the development and delivery of interventions and a focus on social norms as a means of altering individual behaviour. This is more difficult for the prevention of overweight and obesity because the health impact is less apparent, there is a low level of acceptance that the community needs to act, and because social norms around food and physical activity are complex and hard to change [28].

Results from HPC:BAEW and similar CBIs will provide valuable information about the effectiveness of awareness raising and interventions targeted to the individual, when compared with broader strategies such as changes to organisational policies (e.g. healthy eating and physical activity policies in schools and workplaces), creation of strong partnerships and changes to the environment (e.g. bike tracks, provision of water fountains, prompts to use the stairs, availability of healthy food in canteens). There is emerging evidence that these latter approaches are likely to have greater impact at a community level, may be less resource-intensive and are more likely to be sustainable [29].

#### Workforce and community capacity

CBIs require a strategic approach to workforce planning, both within government, in supporting research organisations, and for those employed in communities to deliver programs. In future, this will require a workforce skilled in health promotion, project and change management, evaluation and research, and the ability to utilise evidence to support practice. This requires different skills to those usually employed as policy officers, thus professional development and careful selection of project management staff within government may be necessary. Individuals are unlikely to have all the skills required so careful selection of a team with complementary skills will be important to cover the range required.

In addition to workforce skills, community capacity to adopt a program must be considered before CBIs are implemented [25]. This includes, for example, sufficient resources, adequate skills, aligned values, strong networks and leadership. Experience from HPC:BAEW and other Victorian CBIs shows that communities vary in their capacity to deliver complex projects and to effect behaviour change. A wide range of skills are needed for successful implementation of CBIs: it is therefore important during the planning stage to measure workforce capacity (including skills in partnership building, negotiation, project and change management, program planning and evaluation) and endeavour to balance these skills across all levels of the project [30]. Recruiting and retaining a skilled workforce is challenging, particularly in rural areas and under short-term funding arrangements, so innovative methods of providing support will be required. However, development of workforce capacity will have positive benefits for health promotion interventions in obesity and in other areas, for example tobacco.

There is also large variation in the strength of local partnerships and this needs to be assessed carefully before allocating funding to ensure that resources are invested effectively. Time and resources may be needed to increase community capacity, develop the workforce and strengthen partnerships initially. The availability of tools that will allow measurement of partnerships and organisational capacity is therefore a key issue for this kind of work; several exist, but were not used prior to selection of communities for these Victorian projects. Such gap analysis may assist to tailor the projects and enhance community capacity [25].

### Implications for potential roll-out

By "roll-out" we mean mainstreaming of successful approaches so that a larger proportion of the eligible population can be reached. First and foremost, sufficient planning time is required. Four years from start to finish for a CBI may be too short, especially if it includes planning, comprehensive evaluation and implementation. Extra time may also be needed to allow longer-term follow-up of impact on body mass index (BMI) or other longer-term health outcomes. With regard to interventions, we know that changes at the organisational level and to the environment have widespread impact proportional to the effort put in and are more likely to be sustainable [29]. Therefore they ought to consume a larger proportion of the available resources for CBIs than awareness-raising activities, with individually targeted interventions reserved for high-risk and hard-to-reach groups. CBIs also need to be supported by appropriate regulation, policy, workforce development and social marketing at a state and federal level and continuous effort to change social norms [1] around physical activity, healthy eating and overweight and obesity.

Strong leadership from government is required. Government, with the help of researchers and practitioners, is well placed to advise on suitable interventions so that program staff, with varying skill levels and capacity, do not replicate the work of reviewing evidence across the country. Where there is no, or limited, evidence this needs to be highlighted and a higher-level evaluation built in to allow expansion of the evidence base, a recommendation supported by the Victorian Auditor-General's 2007 report into health promotion [31]. Government (state and federal) is also best placed to lead workforce development, with input from health promotion practitioners, public health staff and the tertiary education sector.

Flexibility is required in allocating funding for CBIs and an output-based approach might be preferable. Potential milestones or outputs to guide funding of CBIs include:

• Demonstration that appropriate partnerships have been developed and are in place.

• Demonstration of adequately skilled project management staff.

• Development of a project plan, including budget, staffing, governance and partnership arrangements, roles and responsibilities, timelines and project objectives and risk management strategy.

• Development of a quality action plan that covers the key interventions as guided by the evidence provided by government.

• Development of a quality evaluation plan guided by government and researchers/evaluators.

• Regular (quarterly) assessment of progress against plans and budget.

At each step, the capacity to stop funding for inadequate progress or to allow more time to meet the milestones would need to be built into service agreements and contracts, supported by a workforce capacity-building strategy to develop essential skills. Involvement of evaluators and experts in CBIs to review and strengthen action and evaluation plans would be one part of such a strategy. Planning for continuation of project manager employment for at least 3 months following expected project completion might also reduce staff turnover and allow for a considered exit and sustainability strategy to be implemented.

A comprehensive evaluation of future projects is challenging on both technical (due to low response rates for measures in individuals) and affordability grounds (if CBIs are "rolled-out" to many more communities). A greater emphasis may be needed on process evaluation complemented by organisation-level measures of impact and monitoring of nutrition and physical activity behaviours. This could occur through state-wide surveys, such as the Victorian Population Health Survey [32] and the Victorian Child Health and Well-being Survey [33], allowing for measurement at a local government area level.

There is currently no Australian monitoring or surveillance system for BMI and experience shows that high response rates are not achievable through opt-in consent, especially for *ad hoc *surveys. For children, opt-out consent, in which participation is assumed unless otherwise indicated, and monitoring through schools (e.g. by school nurses) may be considered [34]—again at a local government area level. For adults, the best option is a survey program conducted by a respected body (e.g. the Australian Bureau of Statistics) but with regular measures (at least every 3-5 years).

Monitoring of impacts on policy, environment, capacity and partnerships could be conducted by the lead agency for the intervention and incorporated as key performance indicators in funding agreements. For all measurement, valid and effective methods would need to be set by government (with input provided by practitioners, researchers and funded agencies)—perhaps in a 'community-based interventions evaluation manual' with supporting training. This would allow consistency of methods across initiatives and be a further means of developing and supporting the workforce. A formal research protocol with a strong study design would then be needed only if there were significant changes in content, process or target groups.

## Conclusions

Community-based interventions offer potential as one of a mix of approaches to preventing obesity. The results of current cost-effectiveness studies will provide important context to the roll-out of obesity prevention projects. Care needs to be taken to incorporate lessons learned from past projects, particularly those related to planning and project management within government, and to allow the flexibility to change course as results emerge from current projects. In the meantime, we should proceed with caution and allow ourselves the flexibility to tailor the process of implementation of CBIs by linking funding to reaching milestones and to the capacity of the selected communities. Strong leadership from government is essential, in partnership with the research community and practitioners, to guide selection of interventions, evaluation of future projects, to enhance monitoring systems for BMI and to support the development of the health promotion workforce.

## Competing interests

All authors are employed by the Victorian Government Department of Health. This department part-funded the six HPC:BAEW projects and approved the final manuscript for publication. Only minor changes were made to the content during this approval process.

## Authors' contributions

MMH, RD and NW conceptualised the paper, drafted sections of the manuscript and provided critical comment on drafts. VM participated in the conceptualisation of the paper and provided critical comment on drafts. All authors read and approved the final manuscript.

## Authors' information

MMH and RD managed the 6 HPC:BAEW projects from May 2008 and provided secretariat support to the HPC:BAEW Project Board. MMH participated as member and later chaired the HPC:BAEW Project Advisory Group. VM chaired the HPC:BAEW Project Board. NW was involved in the Victorian contribution to the federal National Partnership Agreement in Preventive Health "Healthy Workers" and "Healthy Children" initiatives, which include a community-based intervention perspective along with a systems approach to prevention.
